# Improved Efficiency Roll-Off and Operational Lifetime of Organic Light-Emitting Diodes with a Tetradentate Platinum(II) Complex by Using an n-Doped Electron-Transporting Layer

**DOI:** 10.3390/molecules26071835

**Published:** 2021-03-24

**Authors:** Weiqiang Liu, Liang Zhou, Long Yi Jin, Gang Cheng

**Affiliations:** 1 Department of Chemistry, College of Science, Yanbian University, Yanji 133002, China; wqliu@hku.hk; 2 State Key Laboratory of Synthetic Chemistry, HKU-CAS Joint Laboratory on New Materials, Department of Chemistry, The University of Hong Kong, Pokfulam Road, Hong Kong, China; 3 State Key Laboratory of Rare Earth Resource Utilization, Changchun Institute of Applied Chemistry, Chinese Academy of Sciences, Changchun 130022, China; 4 HKU Shenzhen Institute of Research and Innovation, Shenzhen 518053, China; 5 Hong Kong Quantum AI Lab Limited, 17 Science Park West Avenue, Pak Shek Kok, Hong Kong, China

**Keywords:** tetradentate Pt(II) complexes, organic light-emitting diodes, operational lifetime

## Abstract

The efficiency roll-off and operational lifetime of organic light-emitting diodes (OLEDs) with a tetradentate Pt(II) emitter is improved by engaging an n-doped electron-transporting layer (ETL). Compared to those devices with non-doped ETL, the driving voltage is lowered, the charged carrier is balanced, and the exciton density in the emissive layer (EML) is decreased in the device with n-doped ETL with 8-hydroxyquinolinolatolithium (Liq). High luminance of almost 70,000 cd m^−2^ and high current efficiency of 40.5 cd A^−1^ at high luminance of 10,000 cd m^−2^ is achieved in the device with 50 wt%-Liq-doped ETL. More importantly, the extended operational lifetime of 1945 h is recorded at the initial luminance of 1000 cd m^−2^ in the 50 wt%-Liq-doped device, which is longer than that of the device with non-doped ETL by almost 10 times. This result manifests the potential application of tetradentate Pt(II) complexes in the OLED industry.

## 1. Introduction

Organic light-emitting diodes (OLEDs) have attracted significant attention for their potential application in flexible or rollable displays and solid-state lighting sources, attributable to their unique characteristics, such as low power consumption, lightweight, and the ability to be built on flexible or rollable substrates [1,2,3,4]. Phosphorescent emitters are widely utilized in efficient OLEDs to harvest both triplet and singlet excitons for achieving a 100% maximum internal quantum efficiency and high device performance [5,6,7]. Tremendous amounts of effort have been dedicated to improving the performance, especially efficiency, of phosphorescent OLEDs (PhOLEDs) through reducing host singlet-triplet splitting, precisely adjusting host-guest energy gap, and optimizing dopant concentration; the external quantum efficiency (EQE) of current state-of-the-art phosphorescent OLEDs is over 30% [8,9,10]. For instance, Kim and co-workers demonstrated a highly efficient green OLED exhibiting EQE of 32.3% using a green emitter bis(2-phenylpyridine)iridium(III) (2,2,6,6-tetramethylheptane-3,5-diketonate) with high quantum yield and horizontally oriented dipoles. Furthermore, 4,4′,4″-tris(N-carbazolyl)-triphenylamine and bis-4,6-(3,5-di-3-pyridylphenyl)-2-methylpyrimidine were also used as the co-host of the emitting layer (EML) to exploit the exciplex forming character for low driving voltage and good electron-hole balance; [11] Kim and co-workers reported a blue OLED by using an Ir(III) complex with the mpic ancillary ligand as an emitter and the mixture of 3,3-di(9H-carbazol-9-yl)biphenyl (mCBP) and diphenylphosphine-oxide-4-(triphenylsilyl)phenyl as the host in EML. The device showed a high EQE of up to 31.9% and a y-coordinate value lower than 0.2 in the 1931 Commission Internationale de L’Eclairage (CIE) chromaticity diagram [12]. In addition to Pt(II) complexes, high EQEs of over 30% were also achieved in Pt(II) and Pd(II) complexes. Hsu, Escudero, Lee, Chi, and co-workers realized electroluminescence (EL) devices affording intense deep-red/near infrared (NIR) emission with high EQEs of up to 30.4% by exploiting metal-metal ligand charge transfer (MMLCT) transition of square-planar Pt(II) complexes [13]. Li and co-workers showed that a host-free Pd3O8-P yellow-orange OLED emitted light with a peak EQE of 34.8% [14]. In addition to the immediate performance of freshly-fabricated devices, long-term stability is the most crucial criteria to judge the practical application possibility of an OLED. The long-term stability or operational lifetime (LT) of an OLED is mainly determined by the inherent stability of the chemical materials used to fabricate the device [15,16,17]. For PhOLEDs containing a fixed material combination, their LT is strongly influenced by the triplet-triplet annihilation (TTA) and triplet-polaron annihilation (TPA) processes in the emissive layer (EML) due to the long-excitement lifetime of phosphorescent emitters used in these kinds of devices. The TTA and TPA processes generate hot (multiply excited) excitons or polarons, which induce chemical bond dissociation, resulting in an expedited device degradation process [16,17,18,19,20,21]. Therefore, in addition to using stable emitters and functional materials, the design of device structure, in which TTA and TPA processes are minimized, plays a pivotal role in realizing long-term stable OLEDs. Since TTA and TPA processes in a PhOLED are pronounced at conditions of high exciton density and high driving voltage, the adoption of a device structure with a wider exciton recombination zone (ERZ) and lower driving voltage has been demonstrated to be an effective method to improve LT of PhOLEDs [22,23,24,25]. Pin-structure OLEDs, whose hole-transporting layer (HTL) was doped with 2,3,5,6-tetrafluoro-7,7,8,8-tetracyanoquinodimethane and electron transporting layer (ETL) was doped with Cs, were designed and realized by Leo and co-workers [26,27,28]. The doped charge-transporting layers dramatically decreased the device driving voltage and therefore improved the stability of resulting OLEDs [22,28]. Nonetheless, the doping process, especially the n-doping one in pin-structure OLEDs, is quite sophisticated, and a specially designed fabrication facility is needed. Because the hole mobility is typically two magnitudes higher than electron mobility for typical transporting materials for OLEDs, simply increasing the electron mobility of the ETL could decrease the device driving voltage and broaden the ERZ, leading to an improved LT of corresponding OLEDs [29,30]. For instance, KBH_4_ was doped in ETL of an inverted bottom emission OLED as an n-dopant to increase electron injection and reduced the turn-on voltage from 10.1 to 3.0 V [29]. An OLED with n-doped ETL consisting of poly((9,9-dioctylfluorene-2,7-diyl)-alt-(benzo(1–3)thiadiazol-4,7-diyl)) and (pentamethylcyclopentadienyl) (1,3,5-trimethylbenzene)ruthenium dimer was reported recently. The conductivity and luminance of this device have been improved by three and four orders of magnitude when compared to the device with a non-doped ETL [30].

From the chemical structure point of view, tetradentate platinum(II) complexes could be stable phosphorescent emitters due to their “one metal ion + one ligand” design [31]. The lifetime at 50% initial luminance (LT_50_) of over 10,000 h at initial luminance (L_0_) of 100 cd m^−2^ has been reported in OLEDs with tetradentate Pt(II) complexes [32,33]. Since Schmitz and co-workers reported the synthesis of 8-hydroxyquinolinolatolithium (Liq) and used it as interface materials, enabling a decent device performance [34], Liq has been widely used in various organic electronic devices. For instances, solution-processed Liq was used as an effective cathode interfacial layer in inverted perovskite solar cells, leading to a high-power conversion efficiency; [35] Liq mixed with ZnO was used as an efficient electron-injecting layer (EIL) in a polymer light-emitting device, resulting in a lower turn-on voltage and higher efficiency than the device with an EIL fabricated by spin-coated Cs_2_CO_3_ or thermally evaporated Ca [36].

In the present work, we report improvements in efficiency roll-off and long-term stability for PhOLEDs based on the emission of a tetradentate platinum(II) complex by utilizing a Liq-doped ETL. The optimized device displays high luminance of almost 70,000 cd m^−1^ and high current efficiency (CE) of 40.5 cd A^−1^ at high luminance of 10,000 cd m^−2^, which is slightly lower than the maximum CE (41.4 cd A^−1^) of this device by 2.17%. Furthermore, the optimized device with n-doped ETL has an improved LT_50_ of 1945 h at an initial luminance (L_0_) of 1000 cd m^−2^, which is almost ten times longer than that of the device with a non-doped ETL, whose LT_50_ is 202 h at the same L_0_.

## 2. Results and Discussion

**Figure 1:**
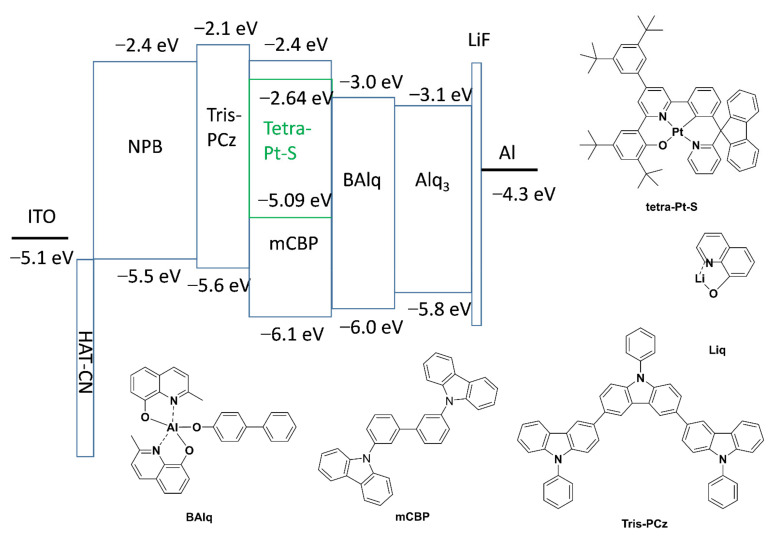
Proposed energy-level diagram of emitting dopant, host and transporting materials; chemical structures of bis(8-hydroxy-2-methylquinoline)-(4-phenylphenoxy)aluminum (BAlq), 3,3-di(9H-carbazol-9-yl)biphenyl (mCBP), 9,9′,9″-triphenyl-9H,9′H,9′H-3,3′:6′,3′-tercarbazole (Tris-PCz), Liq, and tetra-Pt-S.

As depicted in Figure 1, OLEDs were fabricated and characterized with a device structure of indium tin oxide (ITO)/ 1,4,5,8,9,11-hexaazatriphenylene hexacarbonitrile (HAT-CN) (5 nm)/N,N’-bis-(1-naphthalenyl)-N,N’-bis-phenyl-(1,1′-biphenyl)-4,4′-diamine (NPB) (70 nm)/9,9′,9″-triphenyl-9H,9′H,9′H-3,3′:6′,3′-tercarbazole (Tris-PCz) (10 nm)/tetra-Pt-S:mCBP (20 nm)/bis(8-hydroxy-2-methylquinoline)-(4-phenylphenoxy)aluminum (BAlq) (10 nm)/Liq: tris(8-hydroxyquinolinato) aluminum (Alq_3_) (50 nm)/Liq (1 nm)/Al (100 nm). In these devices, the functional layers adjacent to the cathode, a 50-nm-thick Liq-doped Alq_3_, and 1-nm-thick Liq were used as ETL and EIL, respectively. On the side of the anode, a 5-nm-thick layer of HAT-CN was deposited onITO glass substrate as a hole-injecting layer, followed by a 70-nm-thick NPB as a HTL. Furthermore, 10-nm-thick layers of Tris-PCz and BAlq were used as carrier/exciton-blocking HTL and ETL, respectively. Tetra-Pt-S was doped into the 20-nm-thick mCBP as an EML. The chemical structures of Tris-PCz, mCBP, BAlq, Liq, and tetra-Pt-S are also shown in Figure 1.

**Figure 2:**
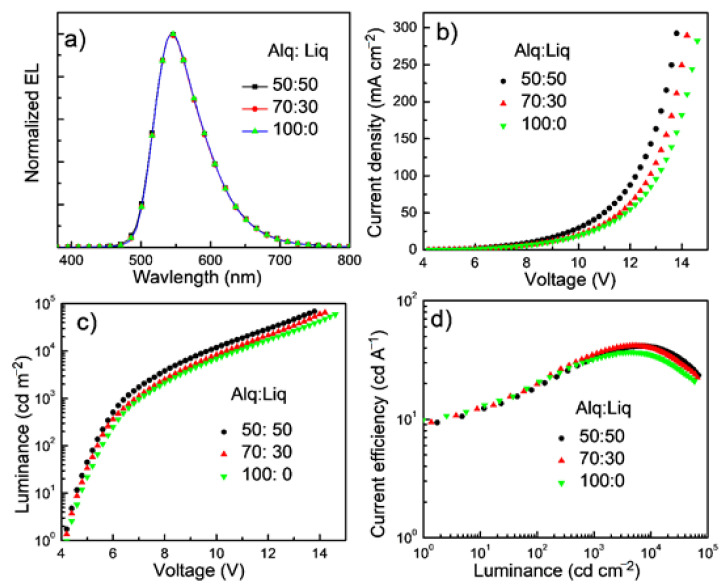
(**a**) Normalized electroluminescence (EL) spectra, (**b**) current density-voltage, (**c**) luminance-voltage and (**d**) current efficiency-luminance characteristics of tetra-Pt-S-based phosphorescent organic light-emitting diodes (PhOLEDs) with different weight ratio of Alq_3_:Liq in electron-transporting layer (ETL).

**Figure 3:**
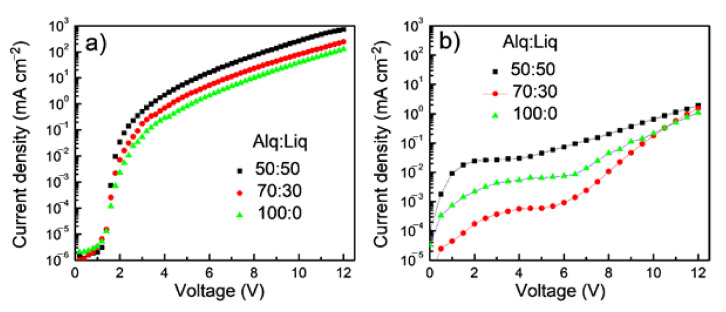
Current density-voltage characteristics of (**a**) electron-only and (**b**) hole-only devices with different weight ratios of Alq_3_:Liq.

**Figure 4:**
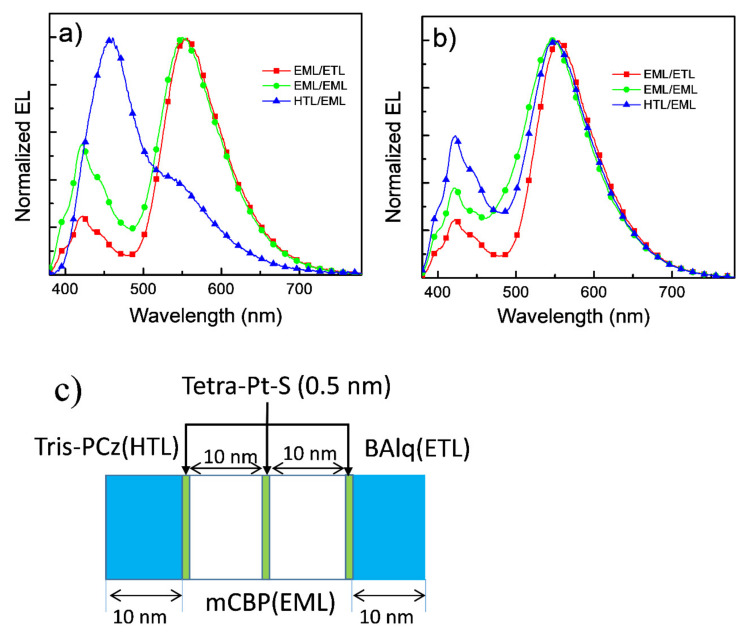
Normalized EL spectra of organic light-emitting diodes (OLEDs) with (**a**) non-doped electron transport layer (ETL) and (**b**) 50%-Liq-doped ETL. A 0.5-nm-thick layer of tetra-Pt-S was inserted in a 20-nm-thick non-doped mCBP emissive layer (EML) at different positions in both devices. (**c**) Schematic diagram of the locations of the tetra-Pt-S layer.

High external quantum efficiency (EQE) of 21.3% and high power efficiency (PE) of 100.2 lm W^−1^ have been achieved in the PhOLED with tetra-Pt-S as the emitting dopant in our previous report [37]. However, the stability (LT_50_ of ~2 h at L_0_ of 4000 cd m^−2^) of this device is not good enough and could be improved by using more stable host and transporting materials. In the current contribution, stable transporting materials, NPB, Tris-PCz, and BAlq, as well as stable host material mCBP, were used to improve the stability of the tetra-Pt-S-based PhOLED [14,32]. Considering the relatively low electron mobility of Alq_3_, Liq was used as an n-dopant to improve the transporting ability of ETL. Normalized EL spectra, current density-voltage, luminance-voltage, and EQE-luminance characteristics of the devices with various doping concentrations of Liq ranging from 30 to 50 wt% in Alq_3_ ETL are depicted in Figure 2a–d, respectively. The device with a non-doped Alq_3_ ETL was also fabricated for comparison. The identical EL spectra (see Figure 2a) indicate that the n-doped ETL hardly affects the emission of tetra-Pt-S in the EML. Both luminance and current density increase with the doping concentration of Liq at a certain driving voltage, attributable to the increased electron-transporting ability of ETL. Electron-only devices (EODs) and hole-only devices (HODs) were fabricated with structures of ITO/Liq (2 nm)/Alq_3_:Liq (100 nm)/Liq (1 nm)/Al (100 nm) and ITO/HAT-CN (10 nm)/Alq_3_:Liq (100 nm)/HAT-CN (10 nm)/Al (100 nm), respectively. The weight ratio of Alq_3_:Liq was 100:0, 70:30, or 50:50 in both EODs and HODs. As depicted in Figure 3, the current density of EODs increases with the ratio of Liq, suggesting that the doping of Liq in Alq_3_ increases the electron-transporting ability of the ETL in the PhOLED with tetra-Pt-S shown in Figure 1. The electron mobility of EODs can be calculated by the Mott–Gurney law assuming both the contacts for injection and extraction are ohmic and the material is defect free and free from energetic disorder [38]. Furthermore, 7.48-fold and 2.68-fold improvements in electron mobility were therefore calculated from Figure 3a for the samples with the Alq_3_:Liq weight ratio of 50:50 and 70:30 when compared with that of the one with pristine Alq_3_, respectively. For HODs, as depicted in Figure 3b, enhanced hole mobilities were also observed upon the doping of Liq at low voltages. However, with the increase of driving voltage, the current densities for all HODs became similar, which is much lower than those of EODs at the same driving voltage. Since the hole mobility of NPB is about 2 magnitudes higher than electron mobility of Alq_3_, hole density in the EML is higher than electron density, causing the ERZ to locate close to the EML/ETL interface in the device with non-doped ETL. For the devices with Liq-doped ETL, the increased electron-transporting ability in ETL increases the electron density in the EML, leading to a more balanced carrier density and broadened ERZ, and in turn, a higher EQE and improved efficiency roll-off at a very high luminance of 10,000 cd m^−2^. To further investigate this phenomenon, we inserted an ultra-thin (0.5 nm) layer of tetra-Pt-S at different positions in the EML, which is a non-doped mCBP in this case, to detect the distribution of ERZ in the devices with and without Liq-doped ETL. As depicted in Figure 4, because of the ultra-thin thickness (0.5 nm) of the tetra-Pt-S layer, the energy transfer from the host mCBP to tetra-Pt-S is not sufficient, and both emissions from tetra-Pt-S and mCBP can be simultaneously observed in all spectra. Nonetheless, the emission ratio of tetra-Pt-S to mCBP at different positions is quite different for the non-doped ETL device. The emission ratio of mCBP/tetra-Pt-S at the EML/ETL interface is similar for both devices with and without Liq-doped ETL. The emission ratio of mCBP/tetra-Pt-S quickly increases with the distance between the ultra-thin layer of tetra-Pt-S to the EML/ETL interface, and the tetra-Pt-S emission almost vanished at the HTL/EML interface of the device with non-doped ETL, suggesting that most excitons formed at the EML/ETL interface in this device. The emission peak located at about 460 nm of the device with the tetra-Pt-S ultra-thin layer at the HTL/EML interface could be the overlap between tetra-Pt-S and mCBP emissions. As depicted in Figure S1, although current density-voltage curves of the device with non-doped ETL were alike for all positions where tetra-Pt-S ultra-thin layer is located, the luminance decreases with the distance between the ultra-thin layer of tetra-Pt-S to the EML/ETL interface at a certain driving voltage due to the relatively low efficiency of the host emission. On the other hand, the emission ratio of mCBP/tetra-Pt-S mildly increases with the distance between the ultra-thin layer of tetra-Pt-S to the EML/ETL interface, and the emission intensity of mCBP is about 60% of that of tetra-Pt-S in the device with 50 wt%-Liq-doped ETL, indicating that the distribution of excitons in more even in this device. As a result, both current density-voltage and luminance-voltage curves of the device with 50 wt%-Liq-doped ETL were similar (See Figure S2). Therefore, improved efficiencies, especially at high luminances of 1000 and 10,000 cd m^−2^, 33.4 and 40.5 cd A^−1^ in current efficiency, and 9.25 and 11.3% in EQE, have been achieved for the device with 50 wt%-Liq-doped ETL (see Table 1). It should be noted in Figure 2d that the efficiency for all devices is relatively low at low luminances, and the maximum efficiency values were achieved at luminances between 1000 and 10,000 cd m^−2^. We attribute the relatively lower efficiency at low luminance to leakage current. As shown in Figure 1, since the highest occupied molecular orbital (HOMO) level of BAlq is higher than that of mCBP, which cannot efficiently block the leaking hole current from the EML. Considering the palpable hole-transporting ability of the ETL at low driving voltages (see Figure 3b), the leakage current could severely lower the device efficiency at low luminance. With increasing driving voltage, the quickly increased electron-transporting ability of the ETL made the hole current ignorable in this layer, and therefore high efficiency was achieved at high luminance of almost 10,000 cd m^−2^. In fact, the EQE of 11.3% in the device with 50 wt%-Liq-doped ETL is even higher than that (11.2%) of the device we previously reported, in which the leakage current was efficiently suppressed by wide-bandgap transporting layers and maximum EQE of 21.3% was achieved [37].

**Figure 5:**
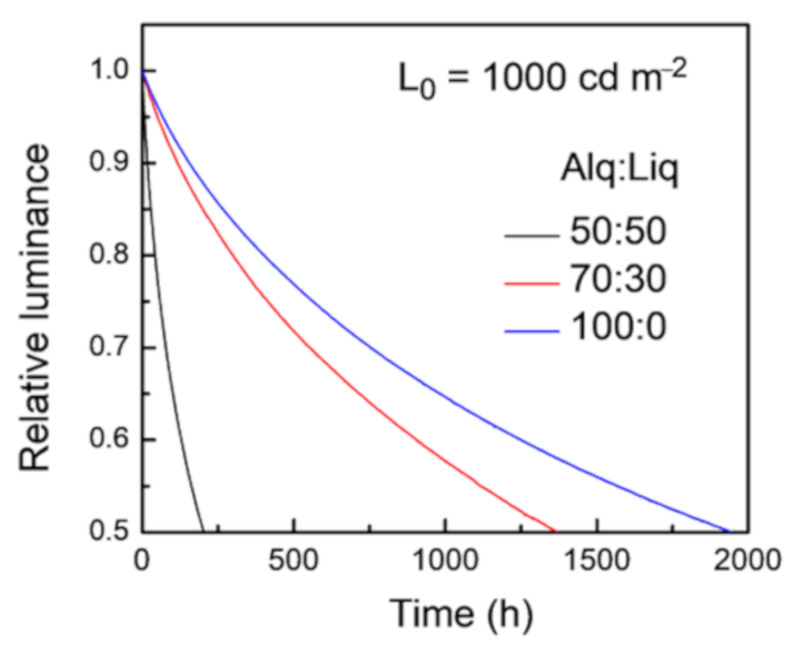
LT of the devices with different weight ratio of Alq_3_:Liq in ETL. The initial luminance is 1000 cd m^−2^ for all devices.

As aforementioned, for PhOLEDs with fixed emitting and supporting materials, their LT is mainly determined by TTA and TPA processes in the EML, which are strongly related to the exciton density in EML. The doped ETL can decrease the driving voltage and broaden the ERZ, which lowers the exciton density in the EML and eventually benefits the LT of the tetra-Pt-S-based OLEDs. Thus, as depicted in Figure 5, LT_50_ of the device with 50 wt%-Liq-doped ETL is 1945 h at L_0_ of 1000 cd m^−2^, which is about 10 times longer than that of the device with non-doped ETL, whose LT_50_ is 205 h at the same L_0_ of 1000 cd m^−2^.

## 3. Materials and Methods

HAT-CN, NPB, mCBP, BAlq, and Liq were obtained commercially and used as received without further purification. Tetra-Pt-S was synthesized following the procedure described in our previous report [37] and purified by gradient sublimation. Indium-tin-oxide (ITO) coated glass with a sheet resistance of 10 Ω/sq was used as the anode substrate. Before film deposition, patterned ITO substrates were cleaned with detergent, rinsed in de-ionized water, acetone, and isopropanol, and finally dried in an oven for 1 h in a cleanroom. The slides were then treated in an ultraviolet-ozone chamber (Jelight, Irvine, CA, USA) for 5 min. The OLEDs were fabricated in a Kurt J. Lesker SPECTROS vacuum deposition system (Kurt J. Lesker, Jefferson Hills, PA, USA) with a base pressure of 10^−7^ mbar. In the vacuum chamber, organic materials were thermally deposited in sequence at a rate of 0.5 Å s^−1^. The doping process in the EMLs was realized using co-deposition technology. Afterward, Liq (1 nm) and Al (100 nm) were thermally deposited at rates of 0.02 and 0.2 nm s^−1^, respectively. The film thicknesses were determined in situ with calibrated oscillating quartz-crystal sensors. Current density-brightness-voltage characteristics, EL spectra, and EQE of EL device were obtained by using a Keithley 2400 (Keithley, Beaverton, OR, USA) source-meter and an absolute external quantum efficiency measurement system (C9920-12, Hamamatsu Photonics, Hamamatsu photonics, Iwata City, Japan). All devices were encapsulated in a 200-nm-thick Al_2_O_3_ thin film deposited by atomic layer deposition (ALD) in a Kurt J. Lesker SPECTROS ALD system (Kurt J. Lesker, Jefferson Hills, PA, USA).

## 4. Conclusions

In summary, we demonstrated an effective strategy to improve efficiency roll-off and LT of PhOLEDs based on the emission of a tetradentate Pt(II) complex tetra-Pt-S by doping Liq into the ETL as an n-dopant. Compared with those of the device with non-doped ETL, the Liq-doped ETL device has increased electron conductivity in the ETL and more balanced carrier density in the EML, leading to a decreased driving voltage and a lower exciton density. Thus, the TTA and TPA processes in the tetra-Pt-S-based OLEDs are restrained, resulting in a longer LT_50_ of 1945 h at L_0_ of 1000 cd m^−2^ and a lower efficiency roll-off of 2.17% at a very high luminance of 10,000 cd m^−2^.

## Figures and Tables

**Table 1:** Key performances of tetra-Pt-S-based PhOLEDs with different weight ratio of Alq_3_:Liq in ETL.

Alq:Liq	EQE (%); CE (cd A^−1^); PE (lm W^−1^) ^a^			Max. Luminance (cd m^−2^)	CIE Coordinates ^b^ (x,y)	LT_50_ (h) ^c^
	Max	at 1000 cd m^−2^	at 10,000 cd m^−2^			
50:50	11.6; 41.4; 16.3	9.25; 33.4; 15.9	11.3; 40.5; 13.0	68,800	0.39, 0.58	1945
70:30	11.7; 41.7; 16.1	9.93; 35.6; 16.0	11.1; 39.8; 12.0	65,400	0.39, 0.58	1371
100:0	10.3; 36.7; 14.3	9.04; 32.4; 14.2	9.71;34.7; 10.1	59,200	0.39, 0.58	202

^a^ External quantum efficiency (EQE); current efficiency (CE); power efficiency (PE); ^b^ at 1000 cd m^−2^; ^c^ at initial luminance (LT) of 1000 cd m^−2^.

## Data Availability

The data presented in this study are available in the article.

## Supplementary Materials

The following are available online at https://www.mdpi.com/1420-3049/26/7/1835/s1, Figure S1: (a) current density-voltage and (b) luminance-voltage characteristics of OLEDs with ultra-thin layer of tetra-Pt-S and non-doped ETL. Figure S2: (a) current density-voltage and (b) luminance-voltage characteristics of OLEDs with ultra-thin layer of tetra-Pt-S and 50%-Liq-doped ETL.

## Sample Availability

Samples of the compounds are not available from the authors.
