# Supplementary material for: Improved Efficiency Roll-Off and Operational Lifetime of Organic Light-Emitting Diodes with a Tetradentate Platinum(II) Complex by Using an n-Doped Electron-Transporting Layer
Source: Molecules. 2021 Mar 24;26(7):1835. doi: 10.3390/molecules26071835 (PMC8037627; doi:10.3390/molecules26071835)
Supplement: Supplementary file 1 [file molecules-26-01835-s001.pdf]

Improved efficiency roll-off and operational lifetime of organic light-emitting diodes with a tetradentate platinum(II) complex by using an n-doped electron-transporting layer

Weiqiang Liu [1,2,3,4], Liang Zhou[3,*], Long Yi Jin[1,*] and Gang Cheng[2,4,5,*]

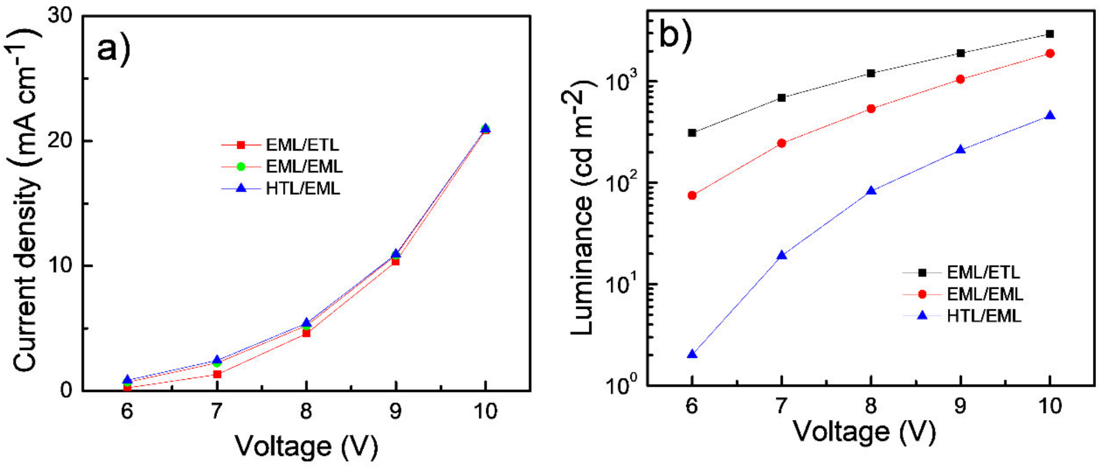

Figure S1. a) current density-voltage and b) luminance-voltage characteristics of OLEDs with ultra-thin layer of **tetra-Pt-S** and non-doped ETL.

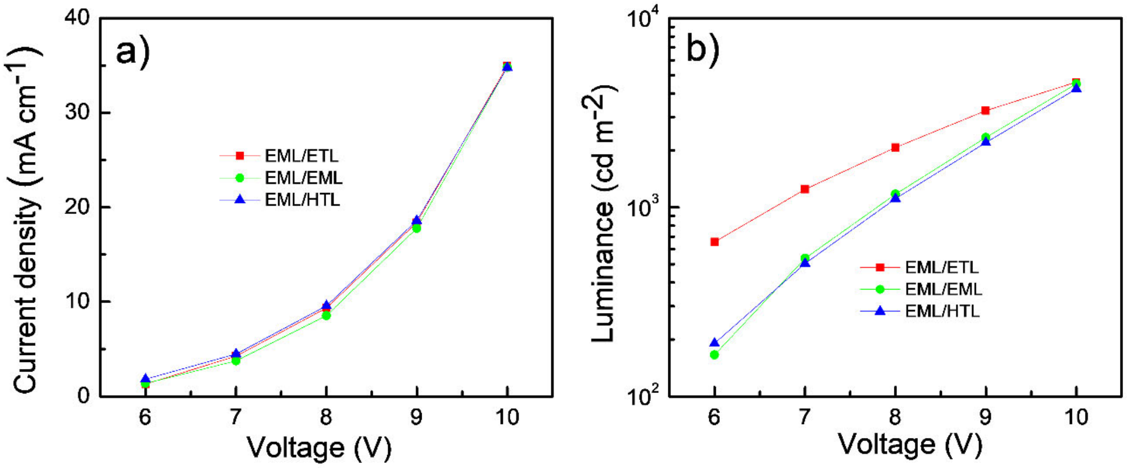

Figure S2. a) current density-voltage and b) luminance-voltage characteristics of OLEDs with ultra-thin layer of **tetra-Pt-S** and 50%-Liq-doped ETL.
